# Recurrent zoonotic *Cryptosporidium parvum* transmission at an animal-contact visitor attraction: insights into infection prevention and control, South West England (2023–2025)

**DOI:** 10.1017/S0950268826101927

**Published:** 2026-07-29

**Authors:** Benjamin Sims, Adrienne Mackintosh, Rachel M. Chalmers, Guy Robinson, Sarah Leaver, Claire E. Brown, Nicholas Young, Chris Brenton, Jennifer Taylor

**Affiliations:** 1 Food Water and Environmental Microbiology Services, Porton, UK Health Security Agency, UK; 2 Department of Bacteriology, Animal and Plant Health Agency, UK; 3 Cryptosporidium Reference Unit, Public Health Wales, Microbiology and Health Protection, Singleton Hospital, UK; 4 Field Service, UK Health Security Agency, UK; 5 Regulatory Services, Cornwall Council, UK; 6 South West Health Protection Team, UK Health Security Agency, UK

**Keywords:** animal-contact visitor attraction, cryptosporidiosis, *Cryptosporidium parvum*, *gp60*, MLVA, One Health, open farm, outbreak, petting farm, zoonoses

## Abstract

Between 2023 and 2025, four outbreaks of zoonotic *Cryptosporidium parvum* infection involving 39 laboratory-confirmed cases occurred at a single animal-contact visitor attraction in South West England. A retrospective outbreak investigation, incorporating elements of a descriptive case series, was undertaken to identify factors associated with repeated transmission and to draw insights for infection prevention and control. The study integrated epidemiological, veterinary, environmental, and molecular data, including exposure questionnaires, animal faecal testing, environmental sampling, observational assessments, and genotyping of human and animal isolates. Zoonotic *gp60* subtype IIaA15G2R1 predominated across outbreaks. In the fourth outbreak, identical *gp60* subtypes and shared multiple-locus variable-number tandem-repeat analysis alleles in lamb and human samples supported zoonotic transmission. Environmental sampling identified faecal indicator organisms and Shiga toxin-producing *E.coli* on high-touch surfaces. An observational assessment identified frequent opportunities for faecal–oral exposure during close contact with young ruminants. Despite compliance with industry standards, persistent environmental contamination, in-pen contact, and child behavioural factors were identified as plausible contributors to transmission. These findings highlight the challenges of preventing zoonotic infection in this setting and support refinement of existing guidance.

## Introduction

Animal-contact visitor attractions are popular leisure destinations in the United Kingdom (UK), drawing millions of visitors each year and contributing substantially to rural tourism and public education [1–3]. These venues bring large numbers of people, particularly families with young children, into close proximity with farm animals, creating well-recognized interfaces for zoonotic transmission [1, 4].

Cryptosporidiosis is a major cause of diarrhoeal disease worldwide and represents a substantial One Health challenge [4–6]. *Cryptosporidium* species are among the most widespread intestinal protozoan parasites, with a global pooled prevalence estimated at approximately 7–8%, disproportionately affecting young children, people living in rural locations, and populations with frequent animal contact [4, 6]. The burden is shared across human and animal health, with *C. parvum* widely recognized as a leading cause of diarrhoea in young ruminants and a dominant zoonotic species [4, 5]. Owing to its combined human, animal, and socioeconomic impact, *Cryptosporidium* has been designated as a neglected disease by the World Health Organization [4, 5].

In England and Wales, cryptosporidiosis is endemic and remains a recurrent cause of outbreaks [1, 7, 8]. *Cryptosporidium* is the most frequently reported protozoal cause of acute gastroenteritis, with several thousand laboratory-confirmed cases reported annually, although the true burden is likely underestimated due to under-ascertainment [4, 8]. National outbreak surveillance indicates that animal contact remains a major transmission pathway [7, 8]. Analysis of outbreaks between 2009 and 2017 identified nearly 200 incidents, with animal-contact settings accounting for a substantial proportion; all such outbreaks were caused by zoonotic *C. parvum* and were strongly seasonal, peaking in the spring [7]. Young ruminants, particularly lambs, were repeatedly implicated, and the same *gp60* subtypes recurred across multiple outbreaks and premises, highlighting the persistent nature of this public health problem [7].

The risks associated with animal-contact settings are amplified by the biological characteristics of *Cryptosporidium.* Young ruminants shed extremely high numbers of oocysts during the neonatal period, often in the absence of clinical disease [9]. Oocysts are immediately infectious, survive for prolonged periods in soil, bedding, water, and on fomites, and are resistant to many commonly used disinfectants [4, 5, 9]. Human infection can occur following ingestion of very low numbers of oocysts, meaning that brief, incidental or indirect contact with contaminated animals, environments or surfaces may be sufficient for transmission [4, 5, 9].

An Industry Code of Practice in England provides guidance intended to reduce zoonotic risks at animal-contact visitor attractions; however, outbreaks linked to these settings continue to occur despite regulatory oversight [2, 8, 10]. A key gap in the literature is therefore understanding why zoonotic transmission persists and which exposure pathways remain incompletely controlled.

Between 2023 and 2025, four discrete outbreaks of *C. parvum* infection were epidemiologically linked to a single animal-contact visitor attraction in South West England. This unusual occurrence of repeated outbreaks within the same setting provided an opportunity to examine recurrent zoonotic transmission over time using an integrated One Health approach.

## Methods

### Hypothesis and study aim

We hypothesized that human cases acquired *C. parvum* infection through direct contact with infected young livestock or indirectly via exposure to faecally contaminated surfaces within the visitor attraction [4, 5, 9, 11, 12]. We further hypothesized that the recurrence of outbreaks reflected persistent opportunities for zoonotic transmission despite existing infection prevention and control measures [5, 6, 11, 12]. Accordingly, the study aimed to identify factors plausibly associated with repeated *C. parvum* transmission, and to draw insights for infection prevention and control in this setting, based on descriptive analysis of outbreak data.

### Study design

A retrospective outbreak investigation, incorporating elements of a descriptive case series, was undertaken using data from four outbreaks epidemiologically linked to a single site (2023–2025). The investigation integrated epidemiological, veterinary, environmental and molecular data to triangulate human, animal, and environmental evidence. Analysis was descriptive; no analytical study was undertaken, and exposure data were summarized without formal comparison between groups or estimation of relative risk. This approach reflected the inability to define a study cohort from the available data. A cohort study may have been feasible with access to detailed booking or visitor records; however, these data could not be extracted by the site operators.

### Veterinary expertise and animal faeces sampling

Veterinary expertise was provided by the Animal and Plant Health Agency (APHA). Remote veterinary input was considered proportionate during the first two outbreaks; however, following the third outbreak, an on-site assessment was undertaken to identify additional measures to reduce transmission risk. Recommendations were provided to the attraction operators and their private veterinary practice.

Animal faecal sampling was not undertaken during the first and second outbreaks, as zoonotic transmission pathways were well recognized and confirmatory evidence of livestock involvement was not considered necessary. Freshly voided faecal material was, however, selectively sampled during the third and fourth outbreaks for testing of *Cryptosporidium* spp. Selection was guided by case-reported animal contact and the recognized role of the species as reservoirs for *C. parvum* [1, 5, 7].

During the third outbreak, faecal samples (*n* = 30) were collected from goats, cows, sheep, rabbits, guinea pigs, pigs, and calves. During the fourth outbreak, faecal samples (*n* = 20) were collected from lamb and goat pens to assess whether these livestock constituted an active source of *C. parvum* during bottle-feeding and petting activities identified as potential risk exposures in this outbreak.

### Diagnostic and reference laboratory testing

Diagnostic testing of human stools was performed using the EntericBio Dx polymerase chain reaction (PCR) gastrointestinal panel [13]. As a notifiable causative agent [14], any *Cryptosporidium* spp. detected was reported to the UK Health Security Agency by the diagnostic microbiology laboratory.

Animal faecal samples were tested at the APHA-accredited parasitology laboratory using the Crypto-Cel IF Test (direct immunofluorescence microscopy) [15, 16].

Positive human and animal samples were referred to the Cryptosporidium Reference Unit (Public Health Wales) for species identification and genotyping [7, 17–20]. In the first instance, species-level confirmation of human specimens was undertaken using real-time PCR assays targeting suitable regions of the Lib13 (*C. parvum*) and A135 (*C. hominis*) genes in a duplex PCR, with the small-subunit rRNA (SSU-rRNA) gene used as required [17]. For animal samples, *C. parvum* was specifically sought as that had been identified as the cause of the outbreaks.

#### gp60 subtyping

Subtyping of *C. parvum* was undertaken by PCR amplification and Sanger sequencing of the hypervariable *gp60* locus [7, 18]. Amplicons were purified and sequenced in the reverse direction in the first outbreak and in both directions in the second, third, and fourth, and chromatograms were examined to generate high-quality consensus sequences. *Gp60* subtypes were assigned by comparison with a reference sequence database, based on the characteristic A-, G-, and R-repeat motifs that define established subtypes [19]. Samples exhibiting reproducible mixed chromatogram peaks within repeat regions were interpreted as mixed infections.

#### Multiple-locus variable-number tandem-repeat analysis (MLVA)

To assess genetic relatedness between *C.* *parvum* from human cases and animal samples, a seven-locus MLVA scheme was applied [20, 21]. This scheme has been validated and implemented as a national reference method for *C.* *parvum* surveillance and outbreak investigations in England and Wales [21]. Loci were amplified using fluorescently labelled primers and amplicons were size-separated by capillary electrophoresis [20]. Alleles were assigned using established locus-specific conversion matrices, with non-amplifying loci recorded as ‘Ø’ [20, 21]. MLVA profiles were expressed as the ordered repeat numbers across all seven loci. Mixed alleles were reported when multiple peaks at a given locus exceeded analytical thresholds and were reproducible across replicate assays; such results were displayed as slash-separated repeat values. The resulting multilocus profiles provided high-resolution discrimination for comparing isolates within and across outbreaks. Integration of MLVA with *gp60* subtyping supported assessment of epidemiological linkage and inference of potential transmission between human and animal samples.

### Case questionnaire and exposure assessment

Local authority teams took primacy for case investigations and administered a retrospective cryptosporidiosis-specific surveillance questionnaire (Supplementary Material) to capture exposures within the 14 days preceding symptom onset. Completed questionnaires were shared with UKHSA for entry into the Case and Incident Management System. Details on the nature and extent of animal contact, including stroking, feeding, kissing, holding, or cuddling, as well as hand hygiene practices, were captured.

### Case definition

A confirmed case was defined as a person with a faecal sample positive for *C. parvum* who had visited the implicated animal visitor attraction within 14 days before symptom onset.

### Retrospective surveillance review

UKHSA reviewed regional surveillance data from 2020 to 2025 to determine the number of animal-contact *C. parvum* outbreaks reported in the region and to identify transferable learning, including from a zoonotic *C. parvum* outbreak linked to an animal-contact event in the South West in 2023 [11].

### Environmental investigations

Local authority outbreak-response visits were undertaken to assess compliance with statutory health and safety requirements and to audit practices against the Industry Code of Practice [2]. To better understand the persistence of transmission despite these measures, a detailed human-factors observational assessment of an in-pen lamb-feeding and petting session was conducted during the fourth outbreak. Attention was focused on behaviours and circumstances that could facilitate faecal–oral transmission, including incidental hand-to-mouth actions, contamination of clothing and footwear, and the adequacy of physical and procedural controls intended to mitigate these risks.

This observation was based on a single session and was not intended to be representative of routine practices across all visits or outbreaks, but rather to provide a descriptive assessment of visitor behaviour and environmental conditions under typical operating conditions to inform risk mitigation.

### Environmental sampling

Environmental surface sampling was not undertaken during the first three outbreaks, as investigations were initiated after visitor exposure had ceased and routine cleaning and disinfection of the animal-contact areas had already been completed prior to site visits. Under these circumstances, environmental sampling was considered unlikely to yield interpretable results or meaningfully inform source attribution.

In addition, these outbreaks were managed as incident responses, with investigative efforts prioritized towards case identification, epidemiological assessment, implementation of control measures, and, where available, animal health intelligence, rather than exploratory environmental microbiology. The lack of contemporaneous access to implicated animals or freshly used visitor-contact surfaces further limited the feasibility and added value of environmental sampling during these earlier incidents.

During the fourth outbreak, environmental sampling was incorporated prospectively as part of a more detailed, hypothesis-driven investigation to better understand persistent transmission under routine operating conditions. Twelve representative environmental swabs were collected from high-frequency touch surfaces within and immediately adjacent to animal-contact areas. Sampling targeted locations with a high likelihood of faecal contamination or frequent hand contact, including metal railings surrounding the petting pens, laminated in-pen seating bales, and hand-wash facilities serving the animal-contact areas.

Samples were obtained immediately after visitors exited the barn following participation in supervised bottle-feeding and petting activities, and before any cleaning took place, thereby maximizing the likelihood of detecting contamination arising from these activities. In addition to surface swabs, a single lamb lap-cuddling blanket, used once during the observed session and laundered beforehand on a hot wash cycle, was collected for faecal indicator testing.

Samples were analysed at the UKHSA Food, Water and Environmental Microbiology Services laboratory using UKAS-accredited methods for the enumeration of Enterobacteriaceae and β-glucuronidase-producing *Escherichia coli* , as specific testing for *Cryptosporidium* spp. on environmental swabs was not operationally feasible [22, 23]. Given the recognized potential for co-circulating zoonotic pathogens in farm environments, and informed by findings from a previous high-profile outbreak [24], additional PCR and culture testing for the detection of Shiga toxin-producing *E.* *coli* (STEC) was undertaken [25], with confirmatory whole-genome sequencing performed at the Gastrointestinal Bacterial Reference Unit, UKHSA Colindale.

## Results

### Case numbers and demographics

**Figure 1.**
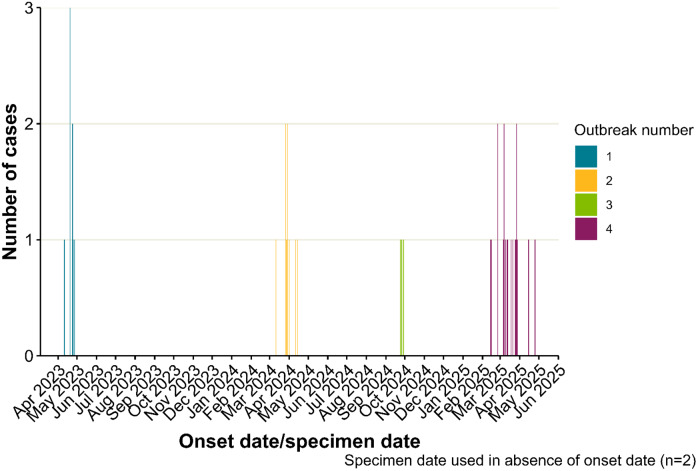
Laboratory-confirmed cases of *C. parvum* associated with the visitor attraction (2023–2025). Figure 1. long description.

**Figure 2.**
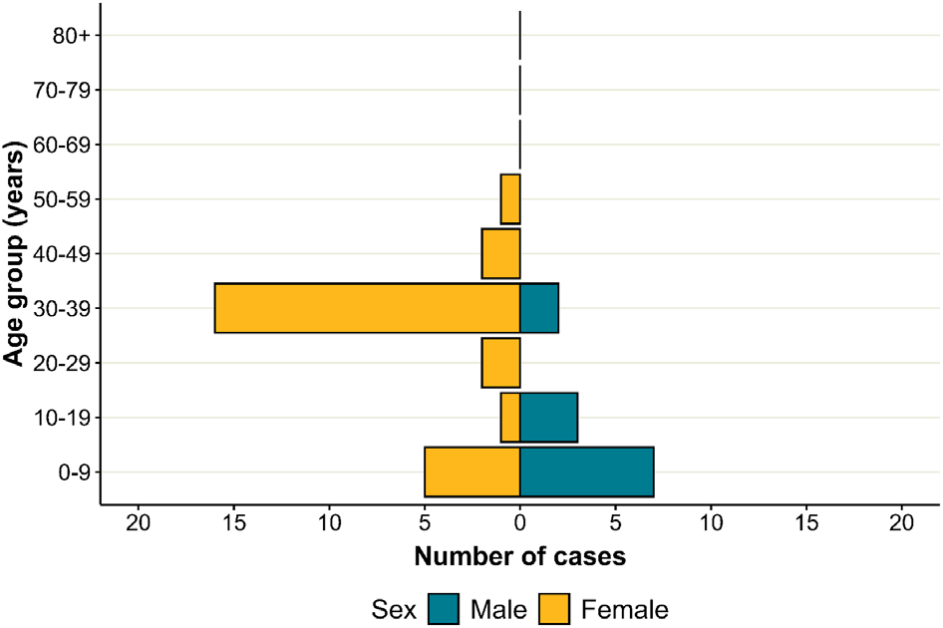
Population pyramid of laboratory-confirmed cases of *C. parvum* associated with the visitor attraction (2023–2025). Figure 2. long description.

A total of 39 laboratory-confirmed primary human cases of *C. parvum* were identified across the four outbreaks (Figure 1). The outbreaks occurred in spring 2023 (*n* = 8), spring 2024 (*n* = 9), autumn 2024 (*n* = 3), and spring 2025 (*n* = 19). Most cases occurred in children under 10 years of age, with approximately equal distribution of boys and girls, and were commonly accompanied by female parents or carers in their 30s (Figure 2). A smaller number of cases were identified among older children and adolescents aged 10–19 years.

### Molecular testing and subtyping

Molecular characterization confirmed that all typed human cases across the four outbreaks were infected with *C. parvum [17]. Gp60* subtyping and MLVA profiles were obtained for most specimens, allowing assessment of genetic relatedness within and between outbreaks (Table 1). Representative *gp60* sequences for each outbreak were submitted to GenBank under accession numbers PZ293349-PZ293356.

**Table 1.** *C. parvum gp60* and MLVA results by outbreak Table 1. long description.

Outbreak	Onset of first and last case	Confirmed cases	*gp60* subtype(s)(*n* = 1 unless otherwise stated)	MLVA profiles(*n* = 1 unless otherwise stated)
1	April–May 2023	8	IIaA15G2R1 (*n* = 6), IIaA16G1R1 IIaA17G1R1	4–14–5–6/7–18/27–28/33–15/16 4–14–5–6/7–18/27–28/33–16 4–14–5–6/7–18/27–33–15/16 4–14–5–6–18/27–33–15/16 4–14–5–6–27–33–15 (*n* = 3) 4–14–5–7–18–28–16
2	March–April 2024	9	IIaA15G2R1 (*n* = 8)	4–14–5–7–27–28–15 (*n* = 8) 4-Ø–5–7–27–28–15
3	September 2024	3	IIaA15G2R1 (*n* = 3)	4–13–5–7–27–29–16 (*n* = 3)
4	March–April 2025	19	Mixed IIaA15G1R1 & IIaA15G2R1 IIaA15G1R1 (*n* = 7) IIaA15G2R1 (*n* = 8)	4–13–5–7–18/27–26/27/29–15/16 4–13–5–7–18/27–26–15/16 4–13–5–7–18/27–25–15 4–13–5–7–18–26–15 (*n* = 2) 4–13–5–7–18–29–15 (*n* = 2) 4–13–5–7–18–29–16 4–13–5–7–27–29–15/16 4–13–5–7–27–25–16 4–13–5–7–27–26–15 (*n* = 2) 4–13–5–7–27–29–15 (*n* = 5)

*MLVA profiles represent the repeat numbers in the seven loci of the validated C.* *parvum* *MLVA scheme. Each integer represents the inferred number of tandem repeats (alleles) at the respective locus. Slash-separated values denote mixed alleles detected at that locus, while ‘Ø’ indicates failure to amplify at that locus.*

#### Outbreak 1

All eight confirmed cases were typed. Three *gp60* subtypes were identified, indicating multiple concurrent circulating strains. MLVA results showed similar diversity: four cases exhibited simple profiles, and four had mixed profiles, with no single dominant MLVA pattern.

#### Outbreak 2

Among the nine cases, eight shared an identical *gp60* subtype and MLVA profile, consistent with a single point source. One case yielded incomplete molecular results (*gp60* non-amplifying; MLVA failure at the second locus), preventing further comparison.

#### Outbreak 3

All three cases shared the same *gp60* subtype and MLVA profile, supporting a single outbreak cluster. Animal sampling for the outbreak yielded no positive results.

#### Outbreak 4

Of the 19 confirmed cases, complete molecular typing was available for 17. Only two *gp60* subtypes were detected, but extensive diversity was seen through multiple simple and mixed MLVA profiles, indicating greater heterogeneity than in outbreaks two and three and the presence of multiple *C. parvum* populations within the same site. In addition to the commonly found IIaA15G2R1 *gp60* subtype that was seen in all the previous outbreaks, IIaA15G1R1 was also detected for the first time at this attraction. The MLVA profiles in the fourth outbreak included some alleles that had been seen in the third.

A lamb faecal sample isolate matched seven human cases by *gp60* subtype and shared alleles at all seven MLVA loci with 17 of the 19 confirmed primary cases (Table 2). A goat faecal sample isolate, although *gp60* PCR-negative, shared six MLVA alleles with the lamb isolate and nine primary confirmed cases (Table 2), supporting epidemiological linkage between livestock and human infection.

**Table 2.** Genetic linkage between human and animal samples in outbreak four Table 2. long description.

Sample type	*gp60* subtype	Alleles present in the MLVA profiles	Linkage to primary confirmed human cases
Human	IIaA15G2R1 IIaA15G1R1	4–13–5–7–18/27–25/26/27/29–15/16	Reference alleles
Lamb faeces	IIaA15G1R1	4–13–5–7–18/27–25/26/29–15	Shares MLVA alleles at all loci with 17 cases
Goat faeces	PCR negative	4-Ø–5–7–27–29–15	Shares 6 MLVA alleles with lamb sample and 9 cases

Ø = No PCR amplification at this locus.

**Table 3.** Extent of close contact by animal type Table 3. long description.

Outbreak	Species involved in visitor contact	Nature of contact reported
1	Lambs, goats, rabbits, guinea pigs	Direct contact with lambs (all cases) Direct contact with goats (most cases) Handling/stroking rabbits and guinea pigs
2	Lambs, goats, sheep, rabbits, guinea pigs	Direct contact with lambs (all cases) Many contacted goats, sheep, rabbits, guinea pigs
3	Calves, goats, sheep, cows, rabbits, guinea pigs	Bottle-feeding and stroking calves Hand-feeding goats and sheep (pellets through rails) Some stroking of cows Handling of rabbits and guinea pigs No lambs present during this period Children’s craft session held inside the same barn as these animals
4	Lambs, goats, rabbits, guinea pigs, pigs, occasional calf contact	Direct contact with lambs (majority of cases) Contact with goats, rabbits, guinea pigs in most cases Limited contact with pigs and some residual calf exposure reported Activities included lamb-feeding/cuddling sessions (observed in environmental assessment)

### Animal contact

Table 3 provides a breakdown of case contact by animal type. All cases interviewed reported direct animal contact. During the first, second, and fourth outbreaks, a total of 36 cases had direct contact with lambs, and 25 additionally reported contact with goats. In the third outbreak, all three cases described close interaction with multiple animal species, including bottle feeding and stroking calves, hand-feeding goats and sheep through railings, and handling rabbits and guinea pigs; lambs were not present during this autumn season. Notably, the third outbreak coincided with children’s craft sessions taking place within the same barn in which calves, goats, rabbits, and guinea pigs were housed, creating additional opportunities for indirect environmental exposure.

### Compliance assessment

Compliance audits by a multi-agency team identified incremental improvements following implementation of recommendations from earlier outbreaks. However, despite these measures, further outbreaks occurred, indicating that residual transmission risks remained.

### Observational findings

An observational assessment of a lamb-feeding and cuddling session confirmed that the recommended controls for designated animal-contact areas were in place [2]; visitors remained outside the pens where the animals were housed, activities were supervised, prior cleaning had occurred, and handwashing facilities were accessible [2]. Nevertheless, several opportunities for faecal−oral transmission were identified, including visitor contact with freshly voided faeces, subsequent contamination of footwear and limited barrier protection afforded by lap blankets. Box 1 summarizes the observational findings documented during the fourth outbreak site visit. These reflect descriptive indicators of potential faecal–oral exposure pathways and should not be interpreted as recommendations.

**Box 1.** Observed behaviours and environmental conditions associated with faecal−oral transmission risk. Box 1. long description.

Faecal exposure inside petting and feeding pen	Freshly voided faeces present in animal petting pen despite prior cleaning Footwear contamination unavoidable inside animal pen Milk bottle dropped on floor and reused without cleaning, potentially touching freshly-deposited faeces on floor Child’s clothes visibly soiled with sawdust from crawling across floor of animal petting pen
Barrier protection	Blankets used for lap cuddling displaced by excitable and fidgety lambs Blankets dropped on floor and reused, potentially touching freshly-deposited faeces on floor
Child behaviour	Toddler crawled on pen floor despite supervision, potentially touching freshly-deposited faeces on floor
Hand hygiene	Parents supervised children’s handwashing but did not always wash their own hands No disinfection of sink taps between uses; taps were not hands-free
Environmental design	Entrance gate (holding pen railings) accessible to children

### Environmental sampling

Enterobacteriaceae were detected in eight of the swabs of the animal-contact area, and *E.coli* was detected in two of the swabs (Table 4). Shiga toxin-producing *E.coli* (STEC) was detected in 5 of the 12 environmental samples and the lamb-cuddling blanket (Table 4). The STEC-positive swabs came from inside the animal-contact area, including the metal pen railings and the seating structure (a plastic-wrapped sawdust bale).

**Table 4.** Results of the environmental (swab) samples taken from the animal petting barns and surrounding areas on 25th March 2025 Table 4. long description.

Sample location	Hygiene indicator tests		Pathogen test	
	*Enterobacteriaceae* mpn/Swab	*E. coli*cfu/swab	STEC PCR	STEC culture and identification
Swab of the lamb hurdle	210	400	Presumptive detection	STEC O5:H- Stx1a EAE + ve
Swab of barn hurdle	2,300	400	Presumptive detection	O unidentifiable: H14 Stx2b EAE ‒ve
Swab of barn hurdle	1,400	<100	Presumptive detection	STEC O5:H- Stx1a EAE + ve
Swab of the plastic-wrapped sawdust bale inside the animal-contact pen	330	<100	Presumptive detection	STEC O5:H- Stx1a EAE + ve
Swab of the barn hurdle – gate	<100	<100	Presumptive detection	Not detected
Swab of the plastic acrylic surrounding the guinea pig enclosure	210	<100	Presumptive detection	STEC O5:H- Stx1a EAE + ve
Swab of the wash hand basin in the animal petting barn	<100	<100	Presumptive detection	Not detected
Swab of the upper barn wash hand basin tap dial	<100	<100	Not detected	–
Swab of the rabbit enclosure	5,700	<100	Not detected	–
Swab of the hurdles of the upper barn	4,600	<100	Not detected	–
Swab of the wash hand basin at the upper barn	210	<100	Not detected	–
Swab of the tap knob at the upper barn	<100	<100	Not detected	–
Blanket (used once during lamb petting/feeding activity)	18,000,000	1,720,000	Presumptive detection	STEC O5:H- Stx1a EAE + ve

A follow-up sampling visit, conducted on a separate occasion immediately after routine post-session cleaning, found no detectable faecal contamination, suggesting that the cleaning procedures were effective when applied promptly.

## Discussion

### Overview of key findings

Over a two-year period, four outbreaks of zoonotic *C. parvum* infection were linked to a single animal-contact visitor attraction, with recurrent transmission associated with close contact between visitors and young ruminants. Molecular, epidemiological, and environmental findings together support the interpretation that each outbreak represented a distinct but internally related transmission event occurring within a shared operational context.

Although each outbreak was discrete, the repeated occurrence of transmission within the same setting suggests the presence of persistent exposure pathways rather than isolated failures. Environmental and observational evidence indicates ongoing opportunities for faecal–oral exposure during routine activities. Taken together, these findings suggest that persistent environmental contamination, in-pen contact with young ruminants and behavioural factors were plausible contributors to transmission, and that existing measures did not fully mitigate exposure risk under routine operating conditions. Given the descriptive nature of the investigation, these findings should be interpreted as indicative of plausible transmission pathways rather than as evidence of causal relationship or relative risk.

### Interpretation of results

The molecular findings indicate a complex pattern across the four outbreaks. Although multiple *gp60* subtypes were detected overall, each outbreak exhibited a dominant subtype and corresponding MLVA profiles consistent with four distinct epidemiological clusters. These observations align with the known diversity of *C. parvum* shed by young ruminants [26] and support the interpretation that the outbreaks were genetically distinct but internally coherent.

Epidemiologically, all cases reported direct contact with young ruminants, and each outbreak coincided with periods when these animals were central to visitor activities. While each outbreak represented a discrete event, the recurrence of cases over a two-year period suggests persistent transmission opportunities rather than isolated failures of practice. This interpretation is supported despite the absence of positive findings from targeted animal sampling during the third outbreak.

Environmental sampling provides additional contextual evidence but should be interpreted with caution. Sampling was limited to a single outbreak and a small number of targeted samples, and did not include direct detection of *Cryptosporidium* spp. Instead, faecal indicator organisms, including Enterobacteriaceae and *E.coli*, were used as proxies for faecal contamination. The detection of these indicators on recently cleaned high-touch surfaces within and adjacent to animal-contact areas, as well as on a laundered lap-cuddling blanket, suggests the presence of ongoing faecal contamination during routine activities, but does not provide direct evidence of pathogen-specific transmission. Taken together, and when considered alongside epidemiological and observational findings, the environmental data are consistent with conditions that may facilitate zoonotic transmission, but it should be interpreted as indicative rather than confirmatory.

### Plausible contributors of repeat transmission

Analysis of the outbreaks suggests that repeated transmission was associated with persistent environmental contamination, in-pen contact with young ruminants, and behavioural factors. Collectively, these conditions impose a sustained operational burden on staff attempting to maintain hygiene and mitigate exposure risks in real time, particularly during seasonal pressures of high visitor throughput.

Young lambs and goat kids can shed high loads of *C. parvum* and defecate frequently, leading to rapid and repeated re-contamination of animal-contact areas [5, 9, 27]. This continual faecal reseeding compromises the effectiveness of routine cleaning and disinfection practices, increases the likelihood that visitors will touch contaminated surfaces, and results in animal hindquarters frequently remaining soiled, thereby sustaining opportunities for faecal–oral exposure.

Observational findings suggested that age-appropriate but risk-unaware behaviours among young children further increased opportunities for exposure, consistent with environmental and molecular findings. For example, children were observed crawling on the floor of the animal-contact area, touching pen railings and inadvertently handling the hindquarters of lambs during lap-cuddling activities.

Even with staff supervision and parental or carer oversight, such behaviours are difficult to prevent or control in real time without fundamentally altering the nature of these activities. Consequently, effective risk reduction relies largely on rigorous hand hygiene and prompt decontamination of clothing and footwear following contact, acting as downstream mitigation rather than primary prevention of exposure [4, 11, 28]. In addition, blankets used during lap-cuddling sessions provided limited and unreliable barrier protection, becoming easily displaced and contaminated despite laundering between sessions, and therefore offered only partial mitigation of direct contact risk.

Taken together, these findings suggest that certain in-pen close-contact activities involving young ruminants represent intrinsically high-risk exposure scenarios that are difficult to mitigate reliably, even when operators comply with the recommendations for animal-contact areas [2, 5, 11, 28]. Understanding these nuances is essential for developing more effective, proportionate approaches.

### Regional outbreak context

A retrospective review of regional surveillance data (2020–2025) identified ten *C. parvum* outbreaks associated with animal-contact visitor attractions across the South West, of which four (40%) occurred at the attraction described in this study. These outbreaks accounted for nearly half of all linked cases during this period, with an even greater proportion observed in 2024–2025.

Although multiple venues reported isolated incidents, only one other attraction experienced more than a single outbreak. This concentration of outbreaks and cases suggests that transmission may persist in a subset of animal-contact settings despite broadly similar regulatory oversight and access to existing guidance.

### Global epidemiology of the IIaA15G2R1 subtype

Across the four outbreaks, the most frequently detected subtype was IIaA15G2R1, a globally widespread *C. parvum* subtype commonly associated with zoonotic transmission [29, 30]. Reviews of *gp60* diversity show that the IIa and IId subtype families dominate *C. parvum* infections in both humans and livestock worldwide, with IIaA15G2R1 among the most frequently reported zoonotic subtypes [29, 30]. Surveillance studies across Europe, including recent national data from Norway, similarly identify IIa and IId as the principal circulating subtype families and recognize IIaA15G2R1 as the predominant *C. parvum* subtype, particularly in settings involving young ruminants and agricultural exposure [29–31].

Recent comparative genomic analyses indicate that IIaA15G2R1 isolates from Europe and the USA cluster within a recently expanded “Western” lineage associated with multiple outbreaks [30, 31]. While the biological basis for this expansion remains incompletely understood, these findings provide a genomic context for its repeated detection in outbreak investigations. The recurrence of IIaA15G2R1 in this study is therefore consistent with broader global and European epidemiological patterns and reinforces the well-established role of young ruminants as major reservoirs for zoonotic *C. parvum* transmission [29–31].

### Interpretation and implications of molecular subtyping

The molecular subtyping added substantial value to the investigation, and its implications extend beyond confirming genetic relatedness [7]. Subtyping demonstrated internal genetic consistency within each outbreak, characterized by the presence of dominant *gp60* subtypes and corresponding MLVA profiles. The second and third outbreaks exhibited uniform *gp60* and MLVA profiles, whereas the first and fourth outbreaks showed greater diversity, with multiple subtypes and mixed MLVA profiles detected. Nevertheless, the overall pattern remained consistent with epidemiologically linked transmission within each outbreak, despite observed subtype and MLVA variability. In the fourth outbreak, identical molecular profiles in human and ruminant faecal samples supported zoonotic transmission.

Detecting multiple *C. parvum* subtypes within or between outbreaks has important implications for how cases are defined in epidemiological investigations [7]. Early reliance on *gp60* results alone may narrow case definitions prematurely, risking the exclusion of true cases infected with less common or initially undetected subtypes. Conversely, the presence of multiple subtypes does not necessarily indicate separate outbreaks, particularly in settings where young ruminants co-shed diverse strains.

Subtype heterogeneity also influences the interpretation of transmission patterns [7]. Mixed profiles, incomplete amplifications, or discordant *gp60*/MLVA results require cautious interpretation, as they may reflect underlying biological diversity rather than multiple infection sources [17, 21, 29, 30]. Once complete human and animal typing data were available, integration of molecular and epidemiological evidence enabled clearer discrimination between outbreaks [21].

Together, these considerations highlight that molecular findings should not be interpreted in isolation but used to inform evolving case definitions and support hypothesis generation during outbreak investigations [7].

### Comparison with previous UK outbreaks

The findings of this study are consistent with evidence from previous UK outbreak investigations demonstrating the challenges of preventing cryptosporidiosis where close contact with young ruminants is permitted [1, 11, 12, 28]. Utsi et al. suggest that infection risk can persist even at venues operating in line with the guidance in place at the time, highlighting the importance of behavioural and environmental factors in exposure [28]. Peake et al. reported significantly increased odds of illness among children who entered lamb pens or engaged in close contact with animals [11]. More recently, Jones et al. provided robust epidemiological evidence that illness risk increases with escalating levels of lamb contact, and that visible faecal contamination of clothing or skin was a strong independent predictor of infection [12].

These findings highlight the difficulty of preventing faecal–oral exposure in settings where animals are handled, held or cuddled, even with supervision and hygiene measures in place. Collectively, this body of evidence suggests that close-contact activities involving young ruminants present inherent control challenges that may not be fully mitigated through existing guidance alone [2, 11, 12, 28].

### Differences in national guidance on close animal contact

Public Health Wales has issued strengthened public-facing advice recommending that visitors to farms avoid close contact with animals, including holding, cuddling, or kissing, due to the increased risk of illness [12, 32, 33]. This advice, which explicitly discourages handling and close interaction with lambs, was reiterated in March 2026 and reported in national media [32, 33]. In contrast, the Industry Code of Practice used in England [2] continues to permit close-contact animal interactions provided certain controls are in place.

This divergence in national advice highlights the challenge of developing proportionate guidance in the absence of applied evidence on the real-world effectiveness of infection prevention and control measures.

### Operational constraints and policy implications

The findings suggest that these inherent exposure pathways limit the effectiveness of existing control measures, and that further risk reduction may be difficult to achieve through procedural controls alone. Many attractions operate under significant operational constraints, including staffing pressures, variable visitor numbers, and peaks in seasonal workload.

Measures such as increased supervision, enhanced cleaning frequency, installation of additional barriers, or upgrades to handwashing facilities can be effective in principle but may be difficult to sustain at the intensity required for highly dynamic, high-contact activities. Smaller or recently established businesses may be particularly constrained in their ability to implement structural or staffing changes without external support.

Close-contact activities, such as lamb lap-cuddling, are often central to the visitor experience and, consequently, to the commercial appeal of these attractions. Restricting or modifying such activities may therefore affect visitor satisfaction, attendance, and financial viability, particularly in seasonal settings.

Operators may be reluctant to adopt more restrictive or less hands-on measures in the absence of clear, consistent national guidance underpinned by robust empirical evidence, while visitors may continue to seek close animal-contact experiences despite risk messaging.

These findings highlight a tension between public health objectives and operational feasibility that cannot be resolved solely through enforcement or improved compliance. Strengthening national guidance may therefore require greater focus on differentiating between activities according to intrinsic risk and systematically evaluating alternative activity designs and behavioural interventions.

Generating applied evidence on what can be delivered under real-world conditions will be essential to support proportionate, implementable policy that reduces zoonotic risk while preserving the educational and economic value of animal-contact visitor attractions. Sector-based support, including training resources, infrastructure improvements, and harmonized public health messaging, may also assist operators in implementing proportionate measures without undermining the viability or attractiveness of their business model.

### Recommendations for further research

This investigation highlights several areas of uncertainty where retrospective outbreak analysis alone is unlikely to provide sufficient resolution. In particular, little is known about the extent to which operators would be willing to alter their business models, or about visitors’ willingness to forgo close-contact activities such as lap-cuddling in favour of safer alternatives. Addressing these gaps is essential for developing evidence-based risk stratification frameworks capable of identifying activities that are acceptably low risk without compromising financial viability.

Future studies should prioritize structured observational and experimental approaches to assess exposure dynamics during routine visitor operations. This includes examining how contamination accumulates and is redistributed within animal-contact areas, how visitors interact with animals, and how these interactions vary by age, supervision, and setting. Generating transferable insights from such work would support more targeted and proportionate recommendations than those achievable through compliance-based inspection alone.

In parallel, further application of *Cryptosporidium* molecular epidemiology could strengthen understanding of transmission dynamics across animal-contact settings. Expanded use of multilocus typing with epidemiological and environmental data would improve understanding of strain diversity, persistence, and reintroduction over time.

Taken together, these approaches would enable a shift from predominantly reactive outbreak investigation towards evidence-based prevention strategies, supporting the development of guidance that is both scientifically robust and practical to implement across diverse animal-contact visitor attractions.

### Limitations

This investigation has several limitations that should be considered when interpreting the findings. Exposure data were derived from retrospective questionnaires and are therefore subject to recall and social-desirability bias, particularly where parents reported on behalf of young children.

No analytical epidemiological assessment was undertaken, as it was not possible to define a study cohort from the available data. Although a cohort study may have been feasible with access to detailed booking or visitor records, IMT members were advised that these data could not be extracted by the site operators. Consequently, exposure data were analysed descriptively without comparison between groups, and it was not possible to estimate relative risks or quantify the relative importance of specific exposures.

Animal faecal sampling was not undertaken during the first two outbreaks, reflecting their management as incident responses rather than prospectively designed research studies. Consequently, there was no contemporaneous microbiological confirmation of livestock involvement in these outbreaks, limiting direct comparison of animal and human data across all incidents. In later outbreaks, the number of animal faecal samples analysed was relatively small, and sampling during the third and fourth outbreaks was selective and risk-based rather than as a comprehensive census of all animals present. These data therefore cannot be used to estimate the prevalence of *C. parvum* infection among animals at the attraction.

Environmental sampling was limited to a single outbreak and a small number of target samples collected during one site visit. As such, these findings cannot be generalized to other time points or conditions and do not provide information on temporal variation or persistence of contamination. In addition, swab samples could not be tested for *Cryptosporidium* spp. and faecal indicator organisms were used as proxies for contamination. As such, these findings support the plausibility of faecal contamination but do not provide direct evidence of pathogen-specific transmission pathways.

Observational assessment of animal-contact activities was restricted to a single lamb-feeding and cuddling session and may not capture the full range of visitor behaviours, staff supervision practices, or environmental conditions that may occur on other occasions.

Molecular typing was incomplete for some human and animal samples, and mixed infections complicated subtype interpretation, necessitating specialist laboratory input.

Finally, as the investigation focused on a single attraction with a specific layout, activity profile, and operational context, the findings may not be fully generalisable to all animal-contact visitor attractions.

## Conclusion

This investigation highlights how in-pen, close-contact activities involving young ruminants can present sustained opportunities for faecal–oral exposure at animal-contact visitor attractions, even where operators implement elements of existing guidance. The findings suggest that persistent environmental contamination, in-pen animal contact, and behavioural factors contribute to ongoing transmission risk under routine operating conditions. These observations underscore the challenges of mitigating zoonotic transmission in such settings and suggest that further refinement of industry guidance, alongside evaluation of alternative activity designs, may be necessary. Future work should focus on developing and assessing practical interventions that reduce exposure while maintaining the educational and operational viability of these attractions.

## Supporting information

10.1017/S0950268826101927.sm001 Sims et al. supplementary material Sims et al. supplementary material

## Data Availability

The data that supports the findings of this study are available on reasonable request to the authors. Restrictions apply to the availability of data, linked to commercial sensitivity of the study location and patient-identifiable information embedded within some of the investigation materials.

## Supplementary material

The supplementary material for this article can be found at http://doi.org/10.1017/S0950268826101927.

## Long descriptions

### Box 1. Long description

The table is organized into five rows of risk categories and their corresponding observations.

* Faecal exposure inside petting and feeding pen: Observations include freshly voided faeces present despite cleaning, unavoidable footwear contamination, a milk bottle dropped on the floor and reused without cleaning, and a child’s clothes soiled with sawdust from crawling.

* Barrier protection: Observations note that blankets used for lap cuddling were displaced by fidgety lambs and that blankets were dropped on the floor and reused.

* Child behaviour: A toddler was observed crawling on the pen floor despite supervision.

* Hand hygiene: Observations indicate that parents supervised children’s handwashing but did not always wash their own hands, and sink taps were not hands-free or disinfected between uses.

* Environmental design: The entrance gate and holding pen railings were noted as being accessible to children.

Navigate back to Box 1..

### Figure 1 Long description

A bar chart with the Y-axis labeled Number of cases, ranging from 0 to 3, and the X-axis labeled Onset date/specimen date, ranging from April 2023 to June 2025 in monthly increments. A legend identifies four outbreaks by color.

* Outbreak 1 (teal): Occurs between April and June 2023. It shows three spikes: one case in April, three cases in May, and two cases in June.

* Outbreak 2 (yellow): Occurs between March and May 2024. It shows one case in March, two cases in April, and one case in May.

* Outbreak 3 (green): A small cluster in October 2024 showing one case.

* Outbreak 4 (purple): The largest temporal cluster, occurring between February and May 2025. It shows one case in February, two cases in March, two cases in April, and one case in May.

A note at the bottom right states: Specimen date used in absence of onset date (n=2).

Navigate back to Figure 1.

### Figure 2 Long description

A population pyramid with the Y-axis representing Age group in years from 0-9 to 80 plus, and the X-axis representing the Number of cases from 0 to 20 on both sides of a central vertical line. The left side (yellow bars) represents Females and the right side (teal bars) represents Males.

* Age 0-9: 5 female cases and 7 male cases.

* Age 10-19: 1 female case and 3 male cases.

* Age 20-29: 2 female cases and 0 male cases.

* Age 30-39: 16 female cases and 2 male cases.

* Age 40-49: 2 female cases and 0 male cases.

* Age 50-59: 1 female case and 0 male cases.

* Age 60-69, 70-79, and 80 plus: 0 cases for both sexes.

The distribution shows a significant outlier in the female 30 to 39 age group, which has the highest number of cases in the entire dataset.

Navigate back to Figure 2.

### Table 1 Long description

The table consists of five columns: Outbreak, Onset on first and last case, Confirmed cases, g p 60 subtype(s), and M L V A profiles.

* Outbreak 1: Occurred April to May 2023 with 8 confirmed cases. Subtypes include I I a A 15 G 2 R 1 (n equals 6), I I a A 16 G 1 R 1, and I I a A 17 G 1 R 1. Six distinct M L V A profiles are listed, including variations with mixed alleles like 4-14-5-6/7-18/27-28/33-15/16.

* Outbreak 2: Occurred March to April 2024 with 9 confirmed cases. Subtype is I I a A 15 G 2 R 1 (n equals 8). Two M L V A profiles are noted, one with a failure to amplify at the second locus (4-null-5-7-27-28-15).

* Outbreak 3: Occurred September 2024 with 3 confirmed cases. Subtype is I I a A 15 G 2 R 1 (n equals 3). The M L V A profile is 4-13-5-7-27-29-16 (n equals 3).

* Outbreak 4: Occurred March to April 2025 with 19 confirmed cases. Subtypes include mixed I I a A 15 G 1 R 1 and I I a A 15 G 2 R 1, with individual counts of 7 and 8 respectively. Ten distinct M L V A profiles are listed, showing high diversity in the fifth and sixth loci, such as 4-13-5-7-27-29-15 (n equals 5).

Navigate back to Table 1.

### Table 2 Long description

The table consists of four columns: Sample type, g p 60 subtype, Alleles present in the M L V A profiles, and Linkage to primary confirmed human cases.

* Row 1: Human sample type. g p 60 subtypes are I I a A 15 G 2 R 1 and I I a A 15 G 1 R 1. M L V A alleles are 4-13-5-7-18/27-25/26/27/29-15/16. These serve as the Reference alleles.

* Row 2: Lamb faeces sample type. g p 60 subtype is I I a A 15 G 1 R 1. M L V A alleles are 4-13-5-7-18/27-25/26/29-15. This sample shares M L V A alleles at all loci with 17 cases.

* Row 3: Goat faeces sample type. g p 60 subtype is P C R negative. M L V A alleles are 4-null-5-7-27-29-15, where null indicates no P C R amplification at that locus. This sample shares 6 M L V A alleles with the lamb sample and 9 cases.

Navigate back to Table 2.

### Table 3 Long description

The table consists of three columns: Outbreak, Species involved in visitor contact, and Nature of contact reported.

* Outbreak 1: Species include lambs, goats, rabbits, and guinea pigs. Contact reported includes direct contact with lambs in all cases, direct contact with goats in most cases, and handling or stroking rabbits and guinea pigs.

* Outbreak 2: Species include lambs, goats, sheep, rabbits, and guinea pigs. Contact reported includes direct contact with lambs in all cases and contact with goats, sheep, rabbits, and guinea pigs in many cases. No calf contact was reported.

* Outbreak 3: Species include calves, goats, sheep, cows, rabbits, and guinea pigs. Contact reported includes bottle-feeding and stroking calves, hand-feeding goats and sheep with pellets through rails, some stroking of cows, and handling of rabbits and guinea pigs. No lambs were present. A children’s craft session was held inside the same barn as these animals.

* Outbreak 4: Species include lambs, goats, rabbits, guinea pigs, pigs, and occasional calf contact. Contact reported includes direct contact with lambs in the majority of cases, contact with goats, rabbits, and guinea pigs in most cases, and limited contact with pigs and residual calf exposure. Activities included lamb-feeding and cuddling sessions.

Navigate back to Table 3.

### Table 4 Long description

The table contains five columns: Sample location, Enterobacteriaceae M P N per Swab, E. coli C F U per swab, S T E C P C R, and S T E C culture and identification.

* Swab of the lamb hurdle: 210 Enterobacteriaceae, 400 E. coli, Presumptive detection, S T E C O 5:H- Stx 1 a E A E positive.

* Swab of barn hurdle: 2,300 Enterobacteriaceae, 400 E. coli, Presumptive detection, O unidentifiable: H 14 Stx 2 b E A E negative.

* Swab of barn hurdle: 1,400 Enterobacteriaceae, less than 100 E. coli, Presumptive detection, S T E C O 5:H- Stx 1 a E A E positive.

* Swab of the plastic-wrapped sawdust bale: 330 Enterobacteriaceae, less than 100 E. coli, Presumptive detection, S T E C O 5:H- Stx 1 a E A E positive.

* Swab of the barn hurdle gate: less than 100 Enterobacteriaceae, less than 100 E. coli, Presumptive detection, Not detected.

* Swab of the plastic acrylic surrounding the guinea pig enclosure: 210 Enterobacteriaceae, less than 100 E. coli, Presumptive detection, S T E C O 5:H- Stx 1 a E A E positive.

* Swab of the wash hand basin in the animal petting barn: less than 100 Enterobacteriaceae, less than 100 E. coli, Presumptive detection, Not detected.

* Swab of the upper barn wash hand basin tap dial: less than 100 Enterobacteriaceae, less than 100 E. coli, Not detected.

* Swab of the rabbit enclosure: 5,700 Enterobacteriaceae, less than 100 E. coli, Not detected.

* Swab of the hurdles of the upper barn: 4,600 Enterobacteriaceae, less than 100 E. coli, Not detected.

* Swab of the wash hand basin at the upper barn: 210 Enterobacteriaceae, less than 100 E. coli, Not detected.

* Swab of the tap knob at the upper barn: less than 100 Enterobacteriaceae, less than 100 E. coli, Not detected.

* Blanket used during lamb petting: 18,000,000 Enterobacteriaceae, 1,720,000 E. coli, Presumptive detection, S T E C O 5:H- Stx 1 a E A E positive.

Navigate back to Table 4.
