# Supplementary material for: Recurrent zoonotic Cryptosporidium parvum transmission at an animal-contact visitor attraction: insights into infection prevention and control, South West England (2023–2025)
Source: Epidemiol Infect. 2026 Jul 29;154:e108. doi: 10.1017/S0950268826101927 (PMC13459008; doi:10.1017/S0950268826101927)
Supplement: Supplementary file 1 [file S0950268826101927sup001.pdf]

Information Classification: CONTROLLED

South West Cryptosporidium Questionnaire

ID Ref No. **Update: April 2024**

Please tick boxes or write in the space(s) provided. **USE BLACK OR DARK BLUE BIRO/PEN.**
The information collected will be kept in strict confidence and will be held in accordance with the Data Protection Act 1998.

Local authority name **Date completed** (dd/mm/yy)
Local Authority Identifier **HPZONE/CIMS Number**
Please complete all sections 1-9 (pages 1 to 6)

Note to EHOs: Additional questions relevant to CIMS XXX begin at question item 6.4 and continue to item 6.19. Please ensure the whole questionnaire is revisited, so that we're not seen to be leading interviewees straight to the farm.

1.1	Name	
1.2	Address	
1.3	Phone number	
1.4	Sex	
1.5	Date of Birth (dd/mm/yy)	
1.6	GP Surgery name	
1.7	GP Surgery address	

2.

2.1	Occupational details	*Tick the appropriate box*<br>Employed ☐ At School ☐ Retired ☐<br>Unemployed ☐ Voluntary/unpaid ☐
2.2	Workplace/School name	*Also include details of any voluntary or unpaid work*<br>*Please put NA if you do not work (paid, unpaid, voluntary) or attend school*
2.3	Workplace/School address	
2.4	Workplace/School postcode	
2.5	Do you do any full time, part time or voluntary work that involves:<br>Handling food Yes ☐ No ☐<br>Caring/teaching children Yes ☐ No ☐<br>Health Care Yes ☐ No ☐	

	Contact with animals	Yes ☐ No ☐

3.1	When did you start to feel ill? (dd/mm/yy)	
3.2	Are you still ill	Yes ☐ No ☐<br><br>If **NO** how many days were you ill?
3.3	Symptoms	Diarrhoea Yes ☐ No ☐<br>*(3 or more loose stools within 24 hours)*<br><br>Blood in stools Yes ☐ No ☐<br><br>Abdominal/Stomach pain Yes ☐ No ☐<br><br>Nausea Yes ☐ No ☐<br><br>Vomiting Yes ☐ No ☐<br><br>Fever Yes ☐ No ☐<br>*(temperature above 37.5ºC)*<br><br>Headaches Yes ☐ No ☐<br><br>Other Yes ☐ No ☐<br><br>Please specify:
3.4 Approx	Did you visit your GP for treatment of your illness? Yes ☐ No ☐	
3.5	Did you visit a hospital for treatment of your illness? Yes ☐ No ☐<br><br>If **YES** which hospital?	
3.6	Were you admitted to hospital for treatment of your illness? Yes ☐ No ☐<br><br>Date of admission (leave blank date not known)? (dd/mm/yy)<br><br>Date of discharge (leave blank date not known)? (dd/mm/yy)<br><br>If exact dates not known, how many days were you in hospital for?	

4.1	Did you come into close contact with anyone else who became ill with similar symptoms in the **14 DAYS** before or after you started to feel unwell? (This includes people within your household and outside, eg work/school contacts).<br><br>Yes ☐ No ☐ If **YES** Please give details in 4.2 below and at end of Page 6 if needed providing details for more than 2 Individuals	
4.2	First and Surname	Contact details (phone number and address)

5.1	Did you spend any nights **OUTSIDE** the UK in the **14 DAYS** before you became ill?<br><br>Yes ☐ No ☐ **if NO go to question 5.2**<br><br>If **YES** please give details of the trip(s) below and on page 6 if further space needed.	
	Date of departure (dd/mm/yy)	
	Date of return (dd/mm/yy)	
	Country visited	
	Town(s)/resorts(s) visited	
	Name of hotel(s)/ campsites visited	
	Name of tour operator if booked through a tour operator	
5.2	Did you spend any nights away from home but **WITHIN** the UK in the **14 DAYS** before you became ill? (Includes staying at friends/relatives, recreational/business trips etc]<br><br>Yes ☐ No ☐ **if NO go to question 6.1**<br><br>If **YES** please give details of the trip(s) below and continue on page 6 if further space needed:	
	Date of departure (dd/mm/yy)	
	Date of return (dd/mm/yy)	
	Place visited (hotel, friend's house etc)	
	Town(s)/village(s) visited	

6.1	Did you have any contact with animals (alive or dead) in the **14 DAYS** before you became ill?<br><br>Yes ☐ No ☐<br>If **YES** what type of animals did you handle?<br><br>*Ask for: cows, goats, sheep, pigs, lambs, poultry, or smaller animals e.g. rabbits, guinea pigs, rodents such as mice or rats*
6.2	Do you have any pets? Yes ☐ No ☐<br><br>If **YES** what type of pet(s)? Cat and number of pets?<br>*(e.g 2 dogs, 3 parrots, 1 goldfish etc)*<br><br>Were any of these pets ill in the **14 DAYS** before you became ill? Yes ☐ No ☐

6.3	Do you live on a farm or small holding? Yes ☐ No ☐
6.4	Did you visit any farms, stables, zoos, visitor attractions, etc. in the **14 DAYS** before you became ill?<br><br>Yes ☐ No ☐<br><br>If **YES** name of where visited?<br><br>If **YES** date of visit?<br><br>If **YES** what areas did you visit while on site?<br><br>Did you handle or touch any animals? Yes ☐ No ☐<br><br>If **YES** what type of animals did you handle? *Ask for: cows, goats, sheep, pigs, lambs, poultry, or smaller animals e.g. rabbits, guinea pigs, rodents such as mice or rats*<br><br>Did you complete any other activities while visiting the farm? Yes ☐ No ☐<br><br>If **YES,** what activities (e.g. play parks, bouncy castles, sand pits, etc.)?
6.5	Did you purchase any food or drink whilst visiting this farm?<br><br>Yes ☐ No ☐<br><br>If **YES,** what food or drink items did you purchase?
6.6	Did you drink any water provided on-site at this farm?<br><br>Yes ☐ No ☐<br><br>If **YES,** where was the water provided?

Questions 6.7-6.9 to be repeated for each animal mentioned by interviewee in Question 6.4.

6.7

Please complete the following for all animals which were interacted with at the farm, stables, zoos, visitor attractions etc.as per Q6.4.

	**Interaction with animal**						
	**Interacted with the animal inside their pen where the animal is kept/housed e.g. the visitor has entered the pen**	**Interacted with the animal in the area where the animal is bottle fed/fed**	**Interacted with the animal outside of their pen – e.g. animal on lap**	**Interacted with the animal through a fence or railing**	**Interacted with the animal in the fields**	**Not sure**	**Did not interact with this animal**
**Lamb**							
**Goat**							
**Cow**							
**Pig**							
**Sheep**							
**Rabbits**							
**Guinea Pigs**							
**Other – please specify**							

6.8 What type of contact did you have with **each animal?**

	Type of contact						
	Touched	Licked by/hand fed the animal	Held on lap/cuddled	Bottle fed	Kissed	Not sure	Did not interact with this animal
Lamb							
Goat							
Cow							
Pig							
Sheep							
Rabbits							
Guinea Pigs							
Other – please specify							

6.9 For those who interacted with the animal **inside** the pen (interacted with the animal inside their pen where the animal is kept/housed e.g. the visitor has entered the pen or interacted with animal in the area where they are bottle fed/fed), did they?:

	Activity inside the pen						
	Sit on the floor/straw	Play with the straw	Eat or drink	Carry in a toy/comforter	Touched the railings	Not sure	Did not interact with this animal
Lamb							
Goat							
Cow							
Pig							
Sheep							
Rabbits							
Guinea Pigs							
Other – please specify							

6.10 Whilst visiting the farm, at what points did you wash their hands (or use alcohol based hand sanitiser)?

	Handwashing					
	Didn't wash hands	Hand sanitizer only	Water only	Soap and water	Soap, water and hand sanitizer	Don't know
Immediately after leaving the small lamb petting shed						
Immediately after contact with any other animals						
Immediately before any food/drink consumption						
When leaving the farm site						

Information Classification: CONTROLLED

6.11 If the person did wash their hands, at any point did they have to wait in a queue for handwashing for longer than a couple of minutes? (rough estimate)

- ☐ No – didn't have to wait
- ☐ Yes – had to wait
- ☐ Not sure
- ☐ Did not wash hands with water at any point

6.12 If the person did wash their hands, what did they typically use to dry their hands?:

- ☐ Did not dry hands
- ☐ Paper towels
- ☐ Hand towel
- ☐ Air hand dryer
- ☐ Not sure
- ☐ Other, please specify:

6.13 Did you visit any other visitor attractions on your visit? Please specify:

7.1 In the **14 DAYS** before you became ill, did you eat any meals or snacks from any parties, receptions or buffets?

Yes ☐ No ☐ If **YES** Please give details of all venues below, continue of page 6 if more space if needed

Venue name & address	Date visited *(dd/mm/yy)*	Dishes eaten

7.2 In the **14 DAYS** before you became ill, did you eat any meals or snacks bought from fast-food outlets? Fast-food outlets include any restaurant, stall or shop **where food is paid for before it is eaten,** eg sandwich bars, burger bars, kebab shops, fish and chip shops, hot dog stands etc.

Yes ☐ No ☐ If **YES** Please give details of all venues below, continue of page 6 if more space if needed .

Venue name & address	Date visited *(dd/mm/yy)*	Dishes eaten

7.3 In the **14 DAYS** before you became ill, did you eat any meals or snacks from any other restaurants, canteen, cafes, pubs or hotels?

Yes ☐ No ☐ If **YES** Please give details of all venues below, continue of page 6 if more space if needed As per note above

Venue name & address	Date visited *(dd/mm/yy)*	Dishes eaten

7.4 Did you eat any of the following other foods in the **14 DAYS** before illness

	**No**	**At home**	**Outside home**	**Where purchased**
**Salad leaves** (e.g. mixed leaves)	☐	☐	☐	

Please specify type and brand:

If YES was it pre-packed/bagged

Yes ☐ No ☐

**Salad vegetables** (e.g. tomatoes)	☐	☐	☐	
Please specify type and brand:				
If YES was it pre-packed/bagged				
Yes ☐ No ☐				
**Other raw vegetables**	☐	☐	☐	
Please specify type and brand:				
If YES pre-packed/bagged				
Yes ☐ No ☐				
	**No**	**At home**	**Outside home**	**Where purchased**
**Fruit**	☐	☐	☐	Occasionally use
Please specify type (e.g. fresh or frozen berries):				
If YES pre-packed/bagged				
Yes ☐ No ☐				
**Pre-packed sandwiches**	☐	☐	☐	

7.5	Did you eat any of the following dairy products in the **14 DAYS before illness**

	**No**	**Yes**	**Type/Brand and Purchased From**
**Cow Milk**	☐	☐	
If YES, was it unpasteurised<br>Yes ☐ No ☐			
**Goats Milk**	☐	☐	
**Cheese**	☐	☐	
**Cream**	☐	☐	
**Yoghurt / Fromage frais**	☐	☐	
**Other dairy e.g. butter, ice cream**<br>Please state type:	☐	☐	

7.7	In the **14 DAYS** before you became ill did you eat any food (including milk) that was bought from:

	**No**	**Yes**	**Name of shop(s)/location**
**Supermarkets**	☐	☐	
**Corner shops**	☐	☐	
**Ethnic groceries**	☐	☐	
**Butchers shops**	☐	☐	
**Milk rounds**	☐	☐	
**Markets**	☐	☐	
**Farm shops**	☐	☐	
**Other**	☐	☐	

7.	Water exposure			
8.1	In the **14 DAYS before illness** did you drink **unboiled water** from:			
		**No**	**Yes**	
**Mains supply**		☐	☐	If **YES** Name of supplier:
**Private supply e.g borehole, well**		☐	☐	If **YES** Name of supplier or source:
**A river, stream or spring**		☐	☐	If **YES** Give location:
**A filter jug**		☐	☐	
**Bottled water**		☐	☐	If **YES** Name of brand:
8.2	In the **14 DAYS** before you became ill did you participate in any of the following activities?			
		**No**	**Yes**	
**Swimming/paddling**		☐	☐	If **YES** Give location:
**Sailing**		☐	☐	If **YES** Give location:
**Canoeing**		☐	☐	If **YES** Give location:
**Windsurfing**		☐	☐	If **YES** Give location:
**Fishing**		☐	☐	If **YES** Give location:
**Other,** please state:		☐	☐	If **YES** Give location:

9.	Additional Information
9.1	In the **14 DAYS** before you became ill did you spend any time outside your usual work or home setting which did not include a night away from home (e.g. visiting the countryside, beaches, parks, playgrounds, day trips etc).<br><br>Yes ☐ No ☐ If YES please give details in space below:
9.2	Please provide any other information you feel is relevant about this illness (foods eaten etc). Including any additional information pertaining to questions asked earlier on:
9.3	On occasion we are required to re-contact individuals to clarify information provided to obtain more details. Would you be happy for us to contact you again for such reasons pertaining to your illness?<br><br>Yes ☐ No ☐

For completion by EHT or HPT: Details of initial advice or action

Thank you for completing this questionnaire
